# HealthyDads.ca: What Do Men Want in a Website Designed to Promote Emotional Wellness and Healthy Behaviors During the Transition to Parenthood?

**DOI:** 10.2196/jmir.7415

**Published:** 2017-10-11

**Authors:** Deborah Da Costa, Phyllis Zelkowitz, Nicole Letourneau, Andrew Howlett, Cindy-Lee Dennis, Brian Russell, Steven Grover, Ilka Lowensteyn, Peter Chan, Samir Khalifé

**Affiliations:** ^1^ Department of Medicine McGill University Montreal, QC Canada; ^2^ Department of Psychiatry Jewish General Hospital Montreal, QC Canada; ^3^ Lady Davis Institute for Medical Research Montreal, QC Canada; ^4^ Faculty of Nursing University of Calgary Calgary, AB Canada; ^5^ Cumming School of Medicine Pediatrics & Psychiatry Alberta Children's Hospital Research Institute for Child and Maternal Health Calgary, AB Canada; ^6^ Department of Psychiatry St. Joseph's Health Centre Toronto, ON Canada; ^7^ Lawrence Bloomberg Faculty of Nursing University of Toronto Toronto, ON Canada; ^8^ Dads Central Ontario Toronto, ON Canada; ^9^ Department of Urology McGill University Health Centre Montreal, QC Canada; ^10^ Department of Obstetrics and Gynecology Jewish General Hospital Montreal, QC Canada

**Keywords:** expectant fathers, mental health, needs assessment

## Abstract

**Background:**

Up to 18% of men experience depression and/or anxiety during the transition to parenthood. Interventions designed specifically to promote the mental health of men during the transition to parenthood are scarce. Internet-delivered interventions may be acceptable and far-reaching in enhancing mental health, parenting knowledge, and healthy behaviors in expectant or new fathers.

**Objective:**

To guide the development of Healthydads.ca, a website designed to enhance mental health and healthy behaviors in expectant fathers, a needs assessment was conducted to identify fathers’ perspectives of barriers to seeking help for emotional wellness, informational needs, and factors affecting the decision to visit such a website.

**Methods:**

One hundred and seventy-four men whose partners were expecting, or had recently given birth, in 3 Canadian provinces (Quebec, Ontario, and Alberta) completed a Web-based survey inquiring about information needs related to psychosocial aspects of the transition to parenthood, lifestyle behaviors, parenting, and factors associated with the decision to visit a father-focused website.

**Results:**

Most men (155/174, 89.1%) reported accessing the Internet to obtain information on pregnancy and spent an average of 6.2 hours online per month. Seeking information about parenting on the Internet was reported by 67.2% (117/174) of men, with a mean of 4.4 hours per month of online searching. Top barriers to seeking help to improve emotional wellness during the perinatal period were: no time to seek help/assistance (130/174, 74.7%), lack of resources available in the health care system (126/174, 72.4%), financial costs associated with services (118/174, 67.8%), and feeling that one should be able to do it alone (113/174, 64.9%). Information needs that were rated highly included: parenting/infant care (52.9-81.6%), supporting (121/174, 69.5%) and improving (124/174, 71.3%) relationship with their partner, work-family balance (120/174, 69.0%), improving sleep (100/174, 57.5%), and managing stress (98/174, 56.3%). Perceiving the website as personally relevant (151/174, 86.8%), credible (141/174, 81.0%), effective (140/145, 80.5%), and having an easy navigation structure (141/174, 81.0%) were identified as important factors related to a first website visit. Providing useful (134/174, 77.0%) and easy to understand (158/174, 90.8%) information, which was also free of charge (156/174, 89.7%), were considered important for deciding to prolong a website visit. Providing the possibility to post questions to a health professional (133/174, 76.4%), adding new content regularly (119/174, 68.4%), and personal motivation (111/174, 63.8%) were factors identified that would encourage a revisit.

**Conclusions:**

Our findings demonstrate that there is substantial interest among expectant and new fathers for using Internet-delivered strategies to prepare for the transition to parenthood and support their mental health. Specific user and website features were identified to optimize the use of father-focused websites.

## Introduction

The transition to parenthood, while a positive and joyful life event for many expectant parents, can also be perceived as a stressful experience that negatively impacts psychological and marital resources for each partner in the couple relationship [1,2]. While numerous studies have been conducted to better understand and promote maternal adjustment during the perinatal period, fewer have targeted expectant fathers. However, expectant and new fathers are also at risk for increased emotional difficulties during the perinatal period. Two meta-analyses have found prevalence estimates between 8.4-10% for paternal antenatal and postpartum depression [3,4]. A recent review suggests that anxiety is also prevalent for men during the prenatal (4.1-16.0%) and postnatal periods (2.4-18.0%) [5]. Paternal emotional difficulties are related to unhealthy lifestyle behaviors (ie, greater use of alcohol and marijuana [6,7]), maternal postpartum depression [3], and a poorer quality and level of fathers’ involvement with their infants [8-10]. Paternal depression occurring during pregnancy or in the early months of the infant’s life may also negatively affect the child’s behavioral, emotional, cognitive, and physical development [11-14]. Despite the prevalence and impact of emotional difficulties during the transition to fatherhood, few expectant or new fathers seek mental health services [15].

A qualitative study conducted to identify the needs of parents during the transition to parenthood found that even though men are now more involved in the antenatal care of their partners than in the past, men reported feeling frustrated by the lack of inclusion, involvement, and information specifically targeting fathers [16]. While this study provides a basis for understanding the gaps in care and needs of men during the transition to parenthood, more comprehensive studies are needed to obtain a better understanding of what men need and prefer, in order to guide and tailor the development of broad-reaching strategies aimed at better preparing men emotionally for the transition to fatherhood.

Despite men indicating a need for information and tools tailored to fathers to better prepare them for the transition, antenatal care and prenatal preparation programs do not systematically address the needs of expectant fathers [17]. In fact, stress management, lifestyle alterations, and relationship changes are often completely omitted from antenatal programs [17]. As a means to disseminate interventions designed to enhance, prevent, and treat mental health, the Internet has enormous appeal as it is anonymous, highly accessible (time and space), sustainable [18], and can be tailored to specific populations or groups [19]. For the majority of people in North America (estimated Internet usage in Canada is 86%) the Internet plays a pivotal role in work, education, and personal domains [20]. As many as 80% of Internet users in developed countries use this resource modality to search for health-related information, typically to find information on conditions, symptoms, and treatments [21,22]. An equally high proportion of men and women are also turning to the Internet to seek information on parenting [23].

A review of online parenting information concluded that many websites remain traditionally gender-biased, with most oriented towards the needs of mothers [23]. The needs of fathers during this life stage may be different from those of mothers. A meta-synthesis of qualitative studies related to early fathering revealed that men felt a strong sense of responsibility as fathers, but felt that they lacked the skills, experience, support, and recognition needed to be fathers [24]. The few studies conducted to date on the needs and knowledge gaps of men during this life stage suggest the importance of targeting fathers prenatally to facilitate their transition to fatherhood, and help them better prepare for the changes and stresses of becoming a parent [24,25]. While a number of Internet sites exist to address concerns related to expectant or new fathers [26,27], there is a lack of published data on their acceptability as a means of disseminating information and their efficacy in enhancing mental health, parenting knowledge, and healthy behaviors in expectant or new fathers.

While the Internet holds promise as a highly accessible and far-reaching mode of disseminating interventions, efficacy trials indicate that the actual uptake and sustained engagement with health interventions are low [28,29]. A sufficient amount of exposure to the intervention content is needed to positively impact the targeted intervention outcomes and initiate behavior change [30,31]. To optimize uptake and sustained use of an electronic health (eHealth) intervention, it is important to identify factors that are related to use in the target population. However, research examining the factors that influence visiting, extending a visit, and revisiting an electronically-delivered intervention remains sparse [32]. Some studies suggest that user (ie, education, age, motivation) and website characteristics (eg, credibility, ease of navigation) influence the use of eHealth interventions, and that the relative importance of these factors may vary depending on phase of exposure (ie, first visit vs revisit) [32]. Features such as the ability to monitor progress in behavior change and provision of tailored feedback have also been shown to increase revisits in studies with other populations [33,34]. Eliciting and incorporating the needs and preferences of the targeted intervention user have been emphasized as critical aspects of eHealth intervention development to optimize the intervention’s usability and acceptability [35,36].

In preparation for the development of an acceptable and feasible evidence-based website (HealthyDads.ca) to enhance mental health and a healthy lifestyle for expectant first-time fathers, a Web-based needs assessment survey was conducted. This needs survey sought to determine: (1) barriers to seeking help to improve emotional wellness; (2) men’s informational needs related to specific topics in the areas of mental health, parenting, and healthy behaviors; and (3) user- and Web-related factors associated with visiting a father-focused website.

## Methods

### Participants and Recruitment

Men were recruited between September 2014 and March 2015 by research staff, or via study flyers in the waiting rooms at university-affiliated obstetrician/gynecologist clinics, ultrasound clinics, and local prenatal classes in the Montreal, Toronto, and Calgary areas. Advertisements about the study were also placed on prenatal and parenting websites [37]. Potential participants were invited to participate in a Web-based survey and informed that the study aim was to learn more about the needs of expectant and new fathers to help the research team develop a new website tailored to men during the transition to parenthood. Eligibility criteria included: ability to understand English or French, partner currently pregnant (>13 weeks gestation) or delivered in the last 6 months, and Internet access.

The study protocol was approved by the McGill University Faculty of Medicine Institutional Review Board and the research ethics committees of the participating institutions (McGill University Health Centre, St. Mary’s Hospital, St. Joseph’s Health Centre, University of Toronto, and University of Calgary). All participants provided informed consent.

Men who indicated an interest in participating in the study were emailed a secure website address (a separate link for each participant) to access the Web-based survey. The survey was available in English and French, and was accessible through Fluid Surveys [38] via a password-protected log-in. Upon entering the log-in identifier number, participants viewed the cover page and a Web-based informed consent page describing the survey, with an option of consenting or declining to continue with the survey. Men who consented were then presented with a series of questions which took approximately 30 minutes to complete. Participants could exit the survey at any time. Upon completion of the Web-based survey, participants received a Can $10 gift card (eg, Amazon) to compensate them for their time.

### Measures

The Needs Assessment Survey asked men to rate the importance and amount of information related to specific psychosocial aspects (ie, depressed mood, stress, work-family balance), parenting (ie, infant care, bonding), and healthy lifestyle behaviors (ie, sleep, physical activity, nutrition) that they would like to access through a Web-based site designed for expectant or new fathers. For items pertaining specifically to pregnancy (ie, *Information to help me learn how to support my partner during pregnancy/labor*), new fathers were asked to respond retrospectively to when their partner was expecting. Each item was rated on a 5-point Likert-type scale with higher scores reflecting greater importance and need for information. The survey also inquired about barriers to seeking help for emotional wellness, with each item rated on a 1 (not a barrier) to 5 (very much a barrier) scale. Using questions adapted from studies by Brouwer and colleagues [31,32,39], men were also queried about user- and Web-related factors associated with expectant fathers visiting a father-focused website. The topics and tools included in the survey were identified from the existing qualitative [16,40] and quantitative literature [23,41-43] (including our own study [44]), as well as the clinical expertise of our team.

Depressive symptomatology was assessed using the Patient Health Questionnaire-2 (PHQ-2). The PHQ-2 consists of 2 of the 9-items from PHQ-9; these include the frequency of depressed mood over the previous 2 weeks [45]. This scale is rated from 0 to 3 where 0= *not at all* and 3=n *early every day*. The validity of this 2-item scale has been verified in studies with men and women, and it is considered a useful tool [45,46].

The 4-Item Perceived Stress Scale (PSS-4) was used to assess perceived stress associated with daily life situations. The reliability and validity of the PSS-4 has been established with women during the perinatal period [47] and in diverse samples that have included men [48]. PSS-4 scores are obtained by reverse coding the positive items and then summing across all four items. Higher scores reflect higher degrees of perceived stress.

In addition to the questionnaires, demographic information (ie, age, marital status, ethnicity, education) was collected, along with monthly duration perusing the Internet to obtain information related to pregnancy and parenting.

### Statistical Analyses

Statistical analyses were performed using the statistical software IBM SPSS version 20.0. The survey data were transferred from Fluid Surveys to SPSS. Descriptive statistics, including means, medians, and standard deviations (SDs) were calculated for all continuous variables, and percentages were calculated for categorical variables. Percent rating >3 for each barrier item (rating scale 1= *not a barrier*; 5= *very much a barrier*) to seeking help for improving emotional wellness was used to identify any barrier endorsed positively, regardless of severity, as very little is known in this area as it relates men during the perinatal period. Percent rating >4 for each item (rated on a 5-point Likert-type scale) related to information importance/amount and website-related usage factors was used to identify topics/factors most strongly needed or preferred, to allow us to best meet the needs of target users while being cognizant of project feasibility and budgetary constraints.

Chi-square tests were conducted to compare level of psychological distress with proportions of: (1) each barrier to improving emotional wellness, (2) preferences for website topics, and (3) factors related to website visits. Participants scoring above the cut-off on the PHQ-2 or in the top quartile on the PSS-4 were classified as distressed for these analyses. These results are presented in Multimedia Appendix 1.

## Results

### Characteristics of Study Participants

Of the 275 men who were eligible and agreed to be emailed a link to the Web-based survey, 203/275 (73.8%) accessed the link to start the survey. Among men accessing the survey, 29/203 (14.3%) did not complete the survey and 174/203 (85.7%) fully completed the survey and comprised the sample that was analyzed. As shown in Table 1, the mean age of our sample was 34.6 years (SD 4.5), with most men (132/174, 75.9%) in the 30-39-year-old age range. The majority of participants (155/174, 89.1%) had a University degree, with only 1.7% (3/174) having a high school diploma or less. Approximately 78.7% (137/174) of the men were Caucasian, and 86.8% (151/174) were employed. Most of the men who completed the survey had partners who were pregnant (141/174, 81.0%) and 19.0% (33/174) had an infant who had recently been born (mean age=11.7 weeks, SD 7.8). Among men whose partner was pregnant at the time of completing the survey, 77.9% (110/141) were expecting their first child.

Most men agreed that it is important to optimize one’s health during their partner’s pregnancy (150/174, 86.2%) to achieve and maintain good health prior to fathering (165/174, 94.8%), and that a father’s eating (166/174, 95.4%) and physical activity patterns (169/174, 97.2%) influence these health behaviors in his children.

**Table 1:** Characteristics of study participants.

Parameter		N=174
Age (years), mean (SD)		34.6 (4.5)
**Education, % (n)**		
	High School or less	1.7 (3)
	Grade 12/Vocational/Technical Program	5.2 (9)
	Some College/University courses	3.4 (6)
	University degree	89.1(155)
**Ethnicity, % (n)**		
	Asian	7.5 (13)
	Black	3.4 (6)
	Caucasian	78.7 (137)
	Other	10.2 (18)
Foreign born, % (n)		32.2 (56)
Married/Cohabitating, % (n)		98.3 (171)
Employment - Working, % (n)		86.8 (151)
**Body mass index (kg/m^2^), % (n)**		
	18.5-25	38.5 (67)
	>25	61.5 (107)
Current Smokers, % (n)		10.3 (18)
**Pregnancy Status, % (n)**		
	Partner currently pregnant	81.0 (141)
	Recently delivered	19 (33)
**Psychosocial, mean (SD)**		
	Patient Health Questionnaire-2	0.61 (1.0)
	4-Item Perceived Stress Scale	4.2 (2.6)

**Table 2:** Barriers to improving emotional wellness during the perinatal period.

Help-Seeking Barriers	n (%)^a^
No time to seek help/ assistance	130 (74.7)
Lack of resources available in the health care system	126 (72.4)
Financial costs associated with services	118 (67.8)
Feeling that one should be able to do it on one’s own	113 (64.9)
Reluctance to talk to others about your moods or anxieties	107 (61.5)
Reluctance from family or friends to talk about emotional aspects of pregnancy/postpartum period	85 (48.9)
Fear that others will judge you	64 (37.0)

^a^Percent rating ≥3 for each barrier statement (1= *not a barrier*; 5= *very much a barrier*).

Most men accessed the Internet from their home (170/174, 97.7%) and 89.1% (155/174) reported accessing it to obtain information on pregnancy, with an average of 6.2 hours per month. Among those accessing Web-based pregnancy information, 42.6% (66/155) reported that the information was helpful. Seeking information about parenting on the Internet was reported by 67.2% (117/174) of men, with a mean of 4.4 hours per month of online searching related to this topic. Only 34.5% (40/117) found the Web-based parenting information helpful. Most users indicated that Web-based information related to pregnancy and parenting was not tailored specifically to fathers (121/154, 78.6%; and 87/117, 74.4%, respectively).

### Psychological Well-Being

The mean score on the PHQ-2 was 0.61 (SD 1.0), with 16.7% (29/174) of participants scoring in the depressed range (score >2). The mean PSS-4 score was 4.2 (SD 2.6), with 26.3% (46/174) of men scoring in the top quartile (score >6) on this stress scale. Self-reported diagnosis of any psychiatric or psychological disorder was 6.9% (12/174). Prior treatment for an emotional problem was reported by 12.6% (22/174) of the sample, with psychotherapy (18/22, 81.8%) and medication (13/22, 59.1%) found as the two most frequently used modalities.

### Barriers for Improving Emotional Wellness

The most frequently endorsed barriers to seeking help to improve emotional wellness for expectant and new fathers during the perinatal period (Table 2) were reported to be lack of time to seek help/assistance (130/174, 74.7%), lack of resources available in the health care system (126/174, 72.4%), financial costs associated with services (118/174, 67.8%), and feeling that one should be able to do it alone (113/174, 64.9%). Chi-square analyses indicated that compared to nondistressed participants, those who were psychologically distressed were more likely to endorse reluctance to talk to others about their moods or anxieties (71.9% vs 56.3%, *P*=.048), and reluctance from family or friends to talk about emotional aspects of pregnancy and the postpartum period (63.2% vs 41.9%, *P*=.008). No group differences were found for the other barriers. These results are detailed in Multimedia Appendix 1.

### Information Needs

Information domains rated in terms of level of importance for a website to enhance emotional wellness, preparing to be a father, and healthy behaviors during the perinatal period (and amount of detail amount needed) are outlined in Table 3. The most highly rated topics were related to: (1) parenting/infant care (52.9-81.6%); (2) partner-oriented issues (66.1-71.3%); and (3) psychosocial topics including their own emotional adjustment, sleep problems, and stress-management (51.2-60.3%). Behavioral topics related to healthy eating and physical activity (42.5-50.6%) were rated as slightly less important. Specific questions in the parenting/infant care and partner-oriented domains that received the highest importance ratings were: how to settle a fussy baby (142/174, 81.6%), information related to baby care (130/174, 74.8%), ways to improve relationship with partner after baby’s birth (124/174, 71.3%), how to support their partner during pregnancy/labor (121/174, 69.5%), balancing work-family life (120/174, 69.0%), and supporting partner to start and maintain breastfeeding (115/174, 66.1%). The top three questions within the psychosocial domain with the highest ratings regarding importance of information were: information about fathers’ emotional adjustment following baby’s birth (105/174, 60.3%), tools to manage sleep problems (100/174, 57.5%), and stress-management tools (98/174, 56.3%).

Chi-square analyses indicated that compared to nondistressed participants, those who were psychologically distressed were more likely to endorse the following topics related to the psychosocial domain: stress-management tools (68.4% vs 50.4%, *P*=.025), strategies to improve mood/emotional well-being (68.4% vs 44.4%, *P*=.003), information about emotional adjustment during their partner’s pregnancy (63.2% vs 45.35, *P*=.027), and access to psychosocial resources (36.6% vs 23.9%, *P*=.045). No other group differences were found for the other topics within the psychosocial domain or questions in the parenting/infant care and partner-oriented domains. These results are detailed in Multimedia Appendix 1.

**Table 3:** The importance of information topics and amount of detailed information needed.

Survey Items	Importance of Information, n (%)^a^	Amount of Information, n (%)^b^
How to settle my baby when s/he is fussy	142 (81.6)	129 (74.2)
Information to increase my knowledge about how to look after my baby	130 (74.8)	121 (69.5)
Information to help me learn how to improve our relationship after pregnancy	124 (71.3)	105 (60.4)
Information to help me learn how to support my partner during pregnancy/labor	121 (69.5)	111 (63.8)
Balancing work-family life	120 (69.0)	95 (54.6)
Ways to support my partner start and maintain breastfeeding	115 (66.1)	90 (51.8)
Information about my emotional adjustment following the baby’s birth	105 (60.3)	79 (45.4)
How to bond with my baby	100 (57.5)	94 (54.1)
Tools to help manage sleep problems	100 (57.5)	81 (46.6)
Information to help me find out about what is offered for dads locally in my area	99 (56.9)	90 (51.7)
Stress-management tools	98 (56.3)	79 (45.4)
How to play with my baby	96 (55.2)	93 (53.4)
Access to parenting resources	94 (54.0)	74 (42.5)
Information to increase confidence in my role as a dad	92 (52.9)	74 (42.6)
Strategies to improve my mood (or emotional well-being)	91 (52.3)	69 (39.7)
Information about my emotional adjustment during my partner’s pregnancy	89 (51.2)	60 (34.5)
Strategies to help me become or stay physically active	88 (50.6)	73 (42.0)
Ways to stay motivated to exercise regularly after my partner has given birth	87 (50.0)	74 (42.6)
Information to help me learn how to cope with this huge change in my life	79 (45.4)	67 (38.5)
Tools to decrease anxiety or fear related to childbirth	76 (43.7)	65 (37.4)
Strategies to help me eat healthy	74 (42.5)	67 (38.5)
Tests to measure my mood/stress levels	66 (37.9)	54 (31.1)
Tips for getting help from my support system	65 (37.3)	50 (28.7)
Access to psychosocial resources	50 (28.7)	39 (22.4)
Information to help me learn more about my feelings about pregnancy	44 (25.4)	42 (24.3)
Chat rooms/social networking with other dads-to-be or new dads	42 (24.1)	31 (17.8)

^a^Percent rating ≥4 for importance of information topics (1= *not at all important*; 5= *very important*). ^b^Percent rating ≥4 for amount of information needed (1= *none*; 5= *detailed*).

**Table 4:** User and website factors reported as important when making a first-visit to a website for expectant fathers to promote their mental health and a healthy lifestyle.

Survey Items			n (%)^a^
**Whether the visitor…**			
	Perceives the website as relevant for himself	151 (86.8)	
	Perceives the source (the organization that provides the intervention) of the website as credible	141 (81.0)	
	Knows that the website is effective	140 (80.5)	
	Is willing to spend time on visiting the website	137 (78.8)	
	Is motivated to visit a father-focused website	131 (75.3)	
	Has access to the Internet at a private location (eg, home, work)	127 (73.0)	
	Gets a positive recommendation about the website	125 (71.8)	
	Wants to improve his mental health and/or lifestyle	112 (64.4)	
	Has positive expectations of father-focused information delivered through the Internet	110 (63.3)	
	Has sufficient skills to use the Internet	73 (42.2)	
	Is referred to the Internet intervention by a health professional (eg, general practitioner, nurse)	67 (38.5)	
	Receives a reminder to visit the website	49 (28.1)	
	Receives an incentive for visiting the website	40 (23.0)	
**Whether the website…**			
	Has a navigation structure that appears to be easy to use at first sight	141 (81.0)	
	Is created by experts in parental well-being behavior change	134 (77.0)	
	Is based on scientific knowledge	131 (75.3)	

^a^Percent rating ≥4 for importance (1= *not at all important*; 5= *extremely important*).

### Factors Influencing Website Usage

The results for features perceived to be very/extremely important for determining whether an expectant father would make a first visit to a website designed to promote their mental health and healthy lifestyle are shown in Table 4. Visitor-related features for a first-time visit that were endorsed as very/extremely important by at least 75% of the sample included: perceiving the website as personally relevant (151/174, 86.8%), credible (141/174, 81.0%), and effective (140/174, 80.5%), as well as user’s willingness (137/174, 78.8%) and motivation (131/174, 75.3%) to spend time visiting the site. Website-related features for determining a first visit that were identified as very/extremely important were reported to be: an easy navigation structure (141/174, 81.0%) and creation by experts in parental well-being and behavior change (134/174, 77.0%).

Table 5 displays the results for features perceived to be very/extremely important for determining whether an expectant father would continue to visit a website long enough to actively engage in, and process, the educational content provided on the website. Visitor-related features for a first-time visit endorsed as very/extremely important by at least 50% of the sample included the user wanting to improve knowledge in relation to the topics (134/174, 77.0%) and experiencing the website as rewarding (129/174, 74.1%). Website-related features for determining whether an expectant father would continue to visit a website long enough to actively engage in and process the educational content provided in the website included: easy to understand information (158/174, 90.8%), free of charge to use (156/174, 89.7%), having useful information for fathers to help them adjust and engage in healthy behavior (147/174, 84.5%), and website attractiveness (123/174, 70.7%).

The results for features perceived to be very/extremely important for determining whether an expectant father revisits a website designed to promote their mental health and healthy lifestyle are shown in Table 6. The most strongly endorsed visitor feature was commitment (111/174, 63.8%), while the most strongly endorsed website-related features were the possibility to post questions to a health professional (133/174, 76.4%) and providing new content on a regular basis (119/174, 68.4%). There were no significant differences in factors influencing website usage by psychological distress status. These results are presented in Multimedia Appendix 1.

**Table 5:** User and website factors reported as important in prolonging a visit to a website for expectant fathers.

Survey Items			n (%)^a^
**Whether the visitor…**			
	Wants to improve his knowledge in relation to the topics of the website	134 (77.0)	
	Experiences the use of the website as rewarding	129 (74.1)	
	Likes receiving (tailored) feedback on the answers he provided on questions	84 (48.3)	
	Knows in advance how long it will take to go through the whole website	46 (26.4)	
	Experiences the use of the website as challenging	36 (20.7)	
**Whether the website…**			
	Provides information that is easy to understand	158 (90.8)	
	Can be used free of charge	156 (89.7)	
	Provides information that is perceived to be useful for dads to help them adjust and engage in healthy behavior	147 (84.5)	
	Is attractive for the visitor to use	123 (70.7)	
	Does not take much time to entirely complete	93 (53.5)	
	Provides brief textual information (ie, does not involve a great deal of reading)	87 (50.0)	
	Displays personal progress through the program (eg, progress bar page numbers)	68 (39.1)	
	Provides testimonials of successes of other dads who used it	66 (37.9)	
	Has a brief registration procedure (eg, the registration of log-in name and password)	63 (36.2)	
	Uses a short questionnaire for providing tailored feedback	62 (35.6)	
	Provides interactive features (eg, tests, forums, games)	57 (32.7)	
	Uses a virtual guide to guide a visitor through the website	41 (23.6)	

^a^Percent rating ≥4 for importance (1= *not at all important*; 5= *extremely important*).

**Table 6:** User and website factors reported as important to revisit a website for expectant fathers.

Survey Items			n (%)^a^
**Whether the visitor…**			
	Is committed to revisiting the website	111 (63.8)	
	Receives a reminder to revisit the website	35 (20.1)	
**Whether the website…**			
	Provides the possibility to post questions to a health professional	133 (76.4)	
	Provides new content on a regular basis	119 (68.4)	
	Uses an approach in which a new visit provides access to all modules or sections in the website	84 (48.2)	
	Provides the possibility for a visitor to monitor his progress in changing a behavior	80 (46.0)	
	Includes the option for the visitor to communicate with others (eg, chat rooms, blogs, forums)	53 (30.5)	
	Uses a modular approach in which a new visit provides access to the next module or section	36 (20.6)	

^a^Percent rating ≥4 for importance (1= *not at all important*; 5= *extremely important*).

## Discussion

Involving potential users in the early stages of intervention development may be key to optimizing the usage, adoption, and impact of eHealth technologies [36]. In preparation for the development of HealthyDads.ca, an electronically-delivered intervention to enhance the mental health and health behaviors of expectant fathers, this study investigated the needs and preferences of fathers towards an eHealth intervention designed to facilitate the transition to fatherhood.

The results of this study showed that expectant and new fathers spend a considerable amount of time on the Internet during their partner’s pregnancy and the postpartum period to search for information on pregnancy (approximately 6 hours per month) and parenting (approximately 4 hours per month). While previous studies have reported on the frequency and patterns of Internet use for information-seeking related to pregnancy and parenting in expectant or new mothers [49-52], this is the first study to document frequency for expectant and new fathers living in Canada. The high use of the Internet as a resource for health-related information during pregnancy and the postpartum period for parents is consistent with findings from the general population [22]. A nationwide survey conducted in the United States found that 72% of Internet users reported searching online for health information of one kind or another within the past year [22]. While our findings support the feasibility of using Web-based educational strategies to reach large numbers of men during the transition to fatherhood, they also point to the need for more father-specific information, given that 3 in 4 men who used the Internet for this purpose reported that the content was not tailored to them.

Consistent with studies conducted with women during the perinatal period, we found that lack of time, lack of resources available in the health care system, and financial costs were common barriers reported by expectant/new fathers (regardless of their level of psychological distress) for seeking help to improve mental health during pregnancy and following the baby’s birth [51,53,54]. The high percentage of expectant and new fathers reporting logistical barriers regarding when and where to obtain services during the perinatal period extends the findings of other Canadian studies with new parents that have examined barriers to accessing support services [55,56]. Delivering an intervention to expectant and new fathers over the Internet may help overcome these logistical barriers, given that Web-based interventions are far-reaching and accessible 24/7, allowing for access at the users’ convenience. Moreover, given the frequency of Internet use by expectant fathers, a Web-based intervention tailored to the needs of men during this life stage may be a highly acceptable mode of delivering evidence-based strategies to promote mental health and better prepare men for fatherhood.

Fathers with elevated psychological distress were more likely to endorse reluctance to talk to others about their moods or anxieties, and reluctance from family or friends to talk about emotional aspects of pregnancy and the postpartum period as barriers to seeking help to improve mental health during pregnancy and following the baby’s birth. It has previously been shown that men at higher risk of depression have more negative attitudes towards help-seeking [57]. These attitudinal barriers may relate to conformity to masculine norm expectations such as self-reliance and resisting displays of vulnerability [58,59]. These findings highlight the importance of incorporating strategies to preempt potential challenges to masculine identities when designing interventions to promote mental health in men, to ensure that they access such resources and remain engaged.

Our findings showed that men want a broad spectrum of Web-based information, including topics related to parenting/infant care (75-82%), supporting (121/174, 69.5%) and improving (124/174, 71.3%) relationships with their partners, work-family balance (120/174, 69.0%), managing stress (98/174, 56.3%), and improving sleep (100/174, 57.5%). The need for Web-based information and strategies to improve mood and coping skills, mobilize social support, and healthy eating were rated as somewhat less important. A study conducted in Australia that offered email and Web-based information tailored to expectant fathers on numerous topics found that fathers were more likely to choose topics related to father-infant interactions [41]. While this finding demonstrates the importance of involved fathering for men, it also suggests that men may be lacking (and therefore actively seeking) practical information on how to engage with their infants in caring and playful activities.

Men reported wanting information on how to support their partner during pregnancy and childbirth, reflecting the importance of an active role that expectant fathers play in their partner’s pregnancy [60]. Uncertainty regarding how to support a pregnant partner has previously been reported by expectant fathers [61]. Increasing fathers’ involvement during pregnancy can make the pregnancy seem more real to expectant fathers [61] and may also lead to better pregnancy outcomes through the reduction of maternal stress [62,63] and depression [64], and support for positive maternal behaviors [65]. Consistent with recent recommendations [40], our findings underscore the importance of including strategies to support their partner during pregnancy when developing father-inclusive antenatal programs. The degree of a father’s involvement during pregnancy is likely influenced by the quality of the relationship with their partner [66], underlying the importance of incorporating strategies such as communication skills training in antenatal programs designed to better prepare couples for the transition to parenthood.

Overall, the informational needs of men in this study centered more on their infant and partner with less emphasis on their own emotional well-being, regardless of their psychological distress level. While these domains are key to the fathering role, they are optimized when fathers themselves are doing well emotionally and feel supported [67,68]. These results may indicate that men feel less of a need for Web-based information on topics related to their mental well-being compared to the parenting and partner domains. Men may also reflect stigma to endorse needs related to psychosocial aspects, or a lack of awareness, concerning the emotional challenges they can experience during the transition to fatherhood. Among the distressed expectant/new fathers in this sample, topics in the psychosocial domain that were most strongly endorsed included stress-management tools and strategies to improve mood/emotional well-being. Considering that subgroups of men struggle with depression, anxiety, and stress during the transition to fatherhood [3-5], father-specific or father-inclusive antenatal programs need to include knowledge and strategies that men can utilize (eg, relaxation techniques, sleep hygiene, physical activity) to optimize emotional adjustment during the transition to fatherhood.

Uptake and sufficient exposure to electronically-delivered interventions remains suboptimal [28,29,69] and requires consideration in the design phase of user- and Web-related factors to optimize usage [31]. The present study is the first to identify potentially important user- and website-specific factors related to uptake and exposure to an Internet-delivered intervention to enhance mental health and a healthy lifestyle in first-time expectant fathers. User-specific factors identified by expectant and new fathers related to making an initial visit to such a website included perceived personal relevance, credibility, effectiveness, time, and motivation. It is not surprising that perceived personal relevance was a top factor that was identified, as men in this study felt that existing websites were not tailored to fathers. Our findings related to the overall user aspects associated with an initial visit are consistent with recommendations made by Brouwer et al [32] who suggested that to impact these user factors, strategies such as targeted promotional information about the electronically-delivered intervention should occur prior to the initial visit to the website, to optimize adoption.

A clear navigation structure and credibility aspects related to the website development by experts with scientific-based content were strongly endorsed as important in deciding to make a first visit. An easy-to-navigate website has consistently been reported by users from all age groups as an important Web-related criterion to determine use [70,71], underscoring the importance of conducting usability testing with users during prototype development. Perceptions related to credibility of a website have been shown to influence judgements related to the quality and usefulness of the information on the website, as well as level of engagement [72,73]. Including a list of scientific resources used to develop the content, expertise of the team (ie, educational and research) contributing to the content of the website with their academic and/or clinical affiliations, as well as funding sources, may help to enhance the credibility of electronically-delivered interventions.

User factors related to wanting to improve knowledge and experiencing the website as rewarding were identified as factors important to prolonging a visit to the website. However, compared to user factors, website-related features were endorsed more strongly as important to prolonging a visit to a father-focused website. A website with easy to understand content, which was free of charge and contained information to help dads adjust and engage in healthy behaviors, with an attractive layout, were factors identified by fathers as important when deciding to prolong a visit. While it has previously been suggested that interactive website features such as tests, forums, and games improve adherence to eHealth interventions targeting mental health and lifestyle promotion [39,74], few respondents in the present study felt strongly about these components.

Fathers in the present study identified the possibility of posting questions to a health professional and regular new content as Web-related features, and personal commitment as a user factor, as being more strongly related to revisiting a website. The use of email reminders to prompt previous users to revisit the website was not highly regarded, even though studies have shown this to be a feature associated with increased usage of electronically-delivered interventions [75,76]. Very few respondents felt that a modular approach, in which a new visit provides access to the next module or section of a website, would entice them to revisit the website. This finding is in line with feedback from users of electronically-delivered interventions targeting expectant mothers [77], as well as those with other populations [78], indicating that users prefer flexibility and access to all modules with some guided features. The user- and Web-related factors identified in relation to uptake and degree of exposure point to the importance of pretesting newly designed Internet-delivered interventions with potential users to ensure that these aspects are optimized prior to the evaluation and implementation phases.

Overcoming barriers to access and strategies to fully engage men with health promotion interventions remains a complex and challenging problem. Identifying the needs of expectant and new fathers, and user- and Web-related factors associated with uptake, are important steps toward optimizing the usability and acceptability of our electronically-delivered program [35,36]. It is important to note that while we surveyed factors that potential users might consider when determining a first-visit, prolonging a visit, and revisiting a website targeting mental health and a healthy lifestyle for expectant fathers, eHealth studies targeting fathers that incorporate these features are needed to determine which of these factors actually contribute to uptake and increased exposure over time in this population. Electronic technology provides a means of creating and disseminating health promotion programs that can be enabled by a variety of channels, including social media, to better reach and engage hard-to-reach groups [79]. Novel ways of engaging men (eg, encouraging men to be agents in helping to promote and connect with other expectant fathers) may also facilitate uptake and use of father-friendly evidence-based electronically-delivered interventions.

### Limitations

Several limitations of the study should be noted. Our survey was distributed to men who had Internet access and may not reflect the needs of men who are not online. However, this is likely to be a small minority given that 95% of Canadians under the age of 55 years have Internet access [20], with 83% having access at home [80]. Our sample size was comprised mostly of Caucasian, highly-educated men, with a high socioeconomic status. Thus, our sample cannot be considered representative of all men in the perinatal period, which limits the generalizability of our findings. The rate of elevated depressed mood in the present sample (29/174, 16.7%) was higher than what has been reported for paternal depression during the perinatal period (8.4%) [4]. It is possible that the responses to the survey are more representative of fathers who are experiencing emotional difficulties during the transition to parenthood. While we surveyed a broad range of user- and Web-related factors previously identified as important in determining a first visit, extending a visit, and revisiting Internet-delivered behavior change interventions [31], other user factors such as anxiety/worry about becoming a father and conformity to masculine gender norms may also influence the level of engagement. While our survey was offered in both English and French languages, the results do not reflect the needs of men who are not fluent in these languages. Given our relatively small sample size, our findings should be considered preliminary. The Can $10 incentive offered following survey completion may have impacted men’s inclination to participate and fully complete the survey. However, findings from Web-based surveys suggest that postpaid survey completion incentives do not substantially increase participation rates [81] or item nonresponse rates [82].

### Implications and Conclusions

We have identified information topics that men find important to include, as well as user and Web-related features, which may enhance exposure to websites targeting fathers. Our findings indicate that during the perinatal period men want Internet-delivered information related to parenting, supporting their partner, and optimizing their emotional adjustment during the transition to parenthood. Gender-tailored elements to reduce stigma and overcome barriers to seeking and accepting help are also important to consider when developing interventions designed to promote mental health in men, such as HealthyDads.ca. Language around mental health and its treatments can itself be viewed as a barrier to engaging men [83]. We have taken steps to ensure that the language in the website is positive and friendly toward men. For example, cognitive-behavioral strategies to reduce mood, stress, and anxiety symptoms are termed *Mental Fitness Tools*. Activity or action-based strategies have also been shown to be useful when working with men [83]. Physical activity interventions, including those that are Web-delivered, have been shown to be acceptable and effective in enhancing mental health and increasing healthy behaviors in men [84-86]. We will provide men with a pedometer and they will have access to Web-based physical activity challenges designed to motivate the user to engage in regular exercise.

Similar to what has been suggested for women [87], a partner’s pregnancy may be a “teachable moment” as men may be more open and interested in interventions designed to promote their own and their family’s well-being and health. The findings from this needs assessment have guided the development of HealthyDads.ca, an evidence-based Internet intervention to enhance mental health and healthy behaviors for expectant first-time fathers. We are currently pilot testing this prototype to determine its acceptability and feasibility, which is an important step to undertake prior to conducting an evaluation of its effectiveness.

## Appendix

Multimedia Appendix 1 Additional tables by psychological distress status.

## Abbreviations

eHealth: electronic health
PHQ: Patient Health Questionnaire
PSS-4: 4-Item Perceived Stress Scale
SD: standard deviation
