# Supplementary material for: HealthyDads.ca: What Do Men Want in a Website Designed to Promote Emotional Wellness and Healthy Behaviors During the Transition to Parenthood?
Source: J Med Internet Res. 2017 Oct 11;19(10):e325. doi: 10.2196/jmir.7415 (PMC5658653; doi:10.2196/jmir.7415)
Supplement: Multimedia Appendix 1 [file jmir_v19i10e325_app1.pdf]

Table 7. Barriers to Improving Emotional Wellness During the Perinatal Period by Psychological Distress Status

Help Seeking Barriers	Distressed (n= 57) %	Not Distressed (n=117) %
No time to seek help/ assistance	77.2	73.5
Lack of resources available in the health care system	77.2	70.1
Financial costs associated with services	71.9	65.8
Feeling that one should be able to do it on one's own	68.4	63.2
Reluctance to talk to others about your moods or anxieties	71.9*	56.4
Reluctance from family or friends to talk about emotional aspects of pregnancy/postpartum	63.2**	41.9
Fear that others will judge you	43.9	33.6

Percent rating ≥ 3 for each barrier statement (1=not a barrier; 5=very much a barrier). Participants who had PHQ-2 scores greater than or equal to 2 or PSS-4 scores greater than or equal to 6 met criteria for distressed. *p<.05; **p<.01

Table 8. The Importance of Information Topics by Psychological Distress Status

	Distressed (n= 57) %	Not Distressed (n=117) %
How to settle my baby when s/he is fussy	87.7	78.6
Information to increase my knowledge about how to look after my baby	82.5	70.9
Information to help me learn how to improve our relationship after pregnancy	77.2	68.4
Information to help me learn how to support my partner during pregnancy/labour	75.4	66.7
Balancing work-family life	71.9	67.5
Ways to support my partner start and maintain breastfeeding	66.7	65.8
Information about my emotional adjustment following the baby's birth	61.4	59.8
How to bond with my baby	64.9	53.8
Tools to help manage sleep problems	59.6	56.4
Stress-management tools	68.4*	50.4
Information to help me find out about what is offered for dads locally in my area	50.9	59.8
How to play with my baby	64.9	50.4
Access to parenting resources	52.6	54.7
Information to increase confidence in my role as a dad	54.1	52.1
Strategies to improve my mood (or emotional well-being)	68.4**	44.4
Ways to stay motivated to exercise regularly after my partner has given birth	54.4	47.9
Strategies to help me become or stay physically active	57.9	47.0
Information about my emotional adjustment during my partner's pregnancy	63.2*	45.3
Information to help me learn how to cope with this huge change in my life	42.1	47.0
Tools to decrease anxiety or fear related to childbirth	50.9	38.2
Strategies to help me eat healthy	38.6	44.4
Tests to measure my mood/stress levels	47.4	33.3
Tips for getting help from my support system	42.1	35.0
Access to psychosocial resources	36.6*	23.9
Information to help me learn more about my feelings about pregnancy	36.8*	19.8
Chat rooms/social networking with other dads-to be or new dads	29.8	21.4

Percent rating ≥ 4 for importance of information topics (1= not at all important; 5= very important); Participants who had PHQ-2 scores greater than or equal to 2 or PSS-4 scores greater than or equal to 6 met criteria for distressed. *p<.05; **p<.01

Table 9. User and Website Factors Reported as Important in Making a First-Visit to a Website for Expectant Fathers to Promote Their Mental Health and a Healthy Lifestyle by Psychological Distress Status

	Distressed (n= 57) %	Not Distressed (n=117) %
**Whether the visitor …..**		
- perceives the website as relevant for himself	84.2	88.0
- perceives the source (the organization that provides the intervention) of website as credible	80.7	81.9
- knows that the website is effective	82.5	79.5
- is willing to spend time on visiting the website	70.2	82.9
- is motivated to visit a father-focused website	68.4	78.6
- has access to the Internet at a private location (e.g., home, work)	64.9	76.9
- gets a positive recommendation about the website	68.4	73.5
- wants to improve his mental health and/or lifestyle	63.2	65.0
- has positive expectations of father-focused information delivered through the Internet	57.9	65.8
- has sufficient skills to use the Internet	32.1	47.0
- is referred to the Internet intervention by a health professional (e.g., GP, nurse)	35.1	40.2
- receives a reminder to visit the website	31.6	26.5
- receives an incentive for visiting the website	21.1	23.9
**Whether the website…..**		
- has a navigation structure that appears to be easy to use at first sight	77.2	82.9
- is created by experts in parental well-being behavior change	75.4	77.8
- is based on scientific knowledge	75.4	74.4

Percent rating ≥ 4 for importance (1= not at all important; 5= extremely important); Participants who had PHQ-2 scores greater than or equal to 2 or PSS-4 scores greater than or equal to 6 met criteria for distressed.

Table 10. User and Website Factors Reported as Important in Prolonging a Visit to a Website for Expectant Fathers by Psychological Distress Status

	Distressed (n= 57) %	Not Distressed (n=117) %
**Whether the visitor …..**		
- wants to improve his knowledge in relation to the topics of the website	70.2	80.3
- experiences the use of the website as rewarding	73.7	74.4
- likes receiving (tailored) feedback on the answers he provided on questions	49.1	47.9
- knows in advance how long it will take to go through the whole website	26.3	26.5
- experiences the use of the website as challenging	24.6	18.8
**Whether the website…..**		
- provides information that is easy to understand	89.5	91.5
- can be used free of charge	87.7	90.6
- provides information that is perceived to be useful for dads help them adjust and engage in healthy behavior	82.5	85.5
- is attractive for the visitor to use	68.4	71.8
- does not take much time to entirely complete	50.9	54.7
- provides brief textual information (i.e., does not involve a lot of reading)	56.1	47.0
- displays personal progress through the program (e.g., progress bar page numbers)	49.1	45.7
- provides testimonials of successes of other dads who used it	36.8	38.5
- has a brief registration procedure (e.g., the registration of login name and password)	38.6	35.0
- uses a short questionnaire for providing tailored feedback	33.3	36.8
- provides interactive features (e.g., tests, forums, games etc.)	38.6	29.9
- uses a virtual guide to guide a visitor through the website	22.8	23.9

Percent rating ≥ 4 for importance (1= not at all important; 5= extremely important); Participants who had PHQ-2 scores greater than or equal to 2 or PSS-4 scores greater than or equal to 6 met criteria for distressed.

Table 11. User and Website Factors Reported as Important to Re-visit a Website for Expectant Fathers by Psychological Distress Status

	Distressed (n= 57) %	Not Distressed (n=117) %
**Whether the visitor …..**		
- is committed to re-visiting the website	66.7	62.4
- receives a reminder to re-visit the website	21.1	19.7
**Whether the website…..**		
- provides the possibility to post questions to a health professional	76.4	79.5
- provides new content on a regular basis	77.2	64.1
- uses an approach in which a new visit provides access to all modules or sections in the website	45.6	49.6
- provides the possibility for a visitor to monitor his progress in changing a behavior	49.1	44.4
- includes the option for the visitor to communicate with others (e.g., chat rooms, blogs, forums)	31.6	29.9
- uses a modular approach in which a new visit provides access to the next module or section	26.3	17.9

Percent rating ≥ 4 for importance (1= not at all important; 5= extremely important); Participants who had PHQ-2 scores greater than or equal to 2 or PSS-4 scores greater than or equal to 6 met criteria for distressed.
